# Neurovascular and infectious disease phenotype of acute stroke patients with and without COVID-19

**DOI:** 10.1007/s10072-022-06133-5

**Published:** 2022-05-23

**Authors:** Simone Beretta, Francesca Iannuzzi, Susanna Diamanti, Elisa Bianchi, Luca D’Urbano, Colella Elisa, Alban Rugova, Carlo Morotti Colleoni, Ettore Beghi, Paolo Bonfanti, Carlo Ferrarese

**Affiliations:** 1 Department of Neurology, San Gerardo Hospital ASST Monza, University of Milano Bicocca, Via Pergolesi 33, 20900 Monza, Italy; 2 NeuroMi (Milan Centre for Neuroscience), Milan, Italy; 3 Department of Infectious Diseases, San Gerardo Hospital ASST Monza, University of Milano Bicocca, Monza, Italy; 4 Department of Neuroscience, Istituto Di Ricerche Farmacologiche Mario Negri IRCCS, Milan, Italy

**Keywords:** COVID-19, Acute stroke, Infectious disease and stroke, Pneumonia, In-hospital mortality

## Abstract

**Background:**

The infectious disease phenotype of acute stroke associated with COVID-19 has been poorly characterized.

**Objective:**

We investigated the neurovascular and infectious disease phenotype of stroke patients with and without COVID-19 infection, and their effect on in-hospital mortality.

**Methods:**

This is a retrospective cohort study of consecutive patients with acute stroke, admitted to any ward of a hub hospital for stroke in Lombardy, Italy, during the first wave of COVID-19. Demographic, neurovascular, infectious disease, and respiratory characteristics were collected. The effect of clinical variables on survival was evaluated using logistic regression models.

**Results:**

One hundred thirty-seven patients with acute stroke were recruited; 30 (21.9%) patients had COVID-19 and represented 2.5% of the 1218 COVID-19 patients hospitalized in the study period. Demographics, comorbidities, stroke type, stroke severity, and etiology did not differ between COVID + stroke patients and non-COVID stroke patients, except for an excess of multi-embolic ischemic stroke in the COVID + group. Most COVID + stroke patients had symptomatic infection (60%) and interstitial pneumonia (70%). COVID + stroke patients required more frequently respiratory support (77% versus 29%; *p* < 0.0001) and had higher in-hospital mortality (40% versus 12%; *p* = 0.0005) than non-COVID stroke patients. Mortality was independently associated with symptomatic interstitial pneumonia (aOR 6.7; 95% CI 2.0–22.5; *p* = 0.002) and, to a lesser extent, with NIHSS on admission (aOR 1.1; 95% CI 1.03–1.2; *p* = 0.007) and recanalization therapies (aOR 0.2; 95% CI 0.04–0.98; *p* = 0.046).

**Conclusion:**

Symptomatic interstitial pneumonia was the major driver of in-hospital mortality in COVID + stroke patients.

**Supplementary Information:**

The online version contains supplementary material available at 10.1007/s10072-022-06133-5.

## Introduction

Coagulopathy is a well-established feature of SARS-CoV-2 infection and its associated disease, COVID-19 [1]. Nonetheless, the relationship between SARS-CoV-2 infection and acute stroke is still unsettled [2, 3]. Several reports consistently showed a reduction of hospital admission for stroke during COVID-19 pandemic, especially in case of transient ischemic attacks and minor strokes, possibly due to fear of coming to hospital and getting infected [4, 5]. However, other studies reported an increased risk of acute ischemic stroke in patients with SARS-CoV-2 infection [6, 7]. Multi-national registries and pooled data analysis reported increased stroke severity and in-hospital mortality and a high proportion of cryptogenic etiology in COVID-associated stroke [8–10].

The infectious disease phenotype of acute stroke associated with COVID-19 has been poorly characterized. Nonetheless, presentations of COVID-19 may critically influence disease course and outcome of COVID + stroke patients.

In this study, we compared the neurovascular and infectious disease features of concurrent acute stroke patients with and without COVID-19 infection, consecutively admitted to a hub hospital for stroke during the first wave of COVID-19 in Lombardy, Italy, and we investigated their effect on in-hospital mortality.

## Methods

### Study design

This is a retrospective cohort study of consecutive patients with acute cerebrovascular diseases (acute ischemic stroke, intracerebral hemorrhage, transient ischemic attack), admitted to any hospital department (acute stroke unit, general medical wards, COVID wards, intensive care unit [ICU], or COVID-ICU) of San Gerardo Hospital, Monza, Lombardy, Italy, a hub hospital for stroke, during the first COVID wave (February 22 to 10 May 2020; 11 weeks).

To be included in the cohort, patients had to be diagnosed with acute cerebrovascular disease by the attending neurologist based on medical history, physical examination, and neuroimaging. Patients with subarachnoid hemorrhage (*n* = 15 in the study period) were excluded. Detailed demographic, neurovascular, infectious disease and respiratory characteristics, date of onset of COVID symptoms, date of onset of stroke symptoms, and in-hospital mortality were collected by review of medical records and compared for patients with and without COVID-19 infection.

### Study definitions

COVID + cases were defined according to the WHO COVID-19 case definition (December 2020) [11], including both confirmed cases of SARS-CoV-2 infection (positive molecular/antigenic virological test ± clinical and epidemiological criteria) and probable cases of SARS-CoV-2 infection (clinical, radiological, and epidemiological criteria; without molecular/antigenic virological testing).

COVID-related symptoms were defined according the WHO COVID-19 case definition as fever, cough, general weakness, fatigue, headache, myalgia, sore throat, coryza, dyspnea, anorexia, nausea, vomiting, diarrhea, anosmia, ageusia, or altered mental status.

Interstitial pneumonia was defined as typical chest imaging findings suggestive of COVID-19, including hazy opacities with peripheral and lower lung distribution on chest radiography and multiple bilateral ground glass opacities with peripheral and lower lung distribution on chest computed tomography (CT).

Transient ischemic attack (TIA) was defined as a transient episode of neurological dysfunction caused by focal brain, spinal cord, or retinal ischemia, without acute infarction [12].

Cryptogenic stroke was defined as acute ischemic stroke of undetermined cause after performing extracranial and intracranial vascular imaging, transthoracic and transesophageal echocardiography, electrocardiography (ECG), ECG telemetry, and hypercoagulable testing or leading to death before completing diagnostic investigations.

Charlson index was used as a well-established summary comorbidity measure associated with short-term mortality [13].

### Statistical analysis

Descriptive statistics were performed for the main demographic and clinical variables. Data were reported as frequencies and percentages for categorical variables, or as medians and interquartile ranges (IQR) for continuous variables. A Kaplan–Meier survival curve was used to describe the probability of occurrence of stroke over time in COVID + patients starting from COVID onset. Selected clinical variables were compared between COVID + and non-COVID patients using the chi-square or the Fisher’s exact test for categorical variables and the Wilcoxon-Mann–Whitney test for continuous variables. Survival probability over time, starting from hospital admission, was compared in COVID + and non-COVID patients using Kaplan–Meier curves and the log-rank test. The effect of selected clinical variables on survival at discharge was evaluated using univariable and multivariable logistic regression models. The choice of variables to be included in both univariable and multivariable analyses was based on clinical reasons. Given the small sample size, we decided to analyze variables that could be considered as potential risk or protective factors for in-hospital mortality in stroke patients. The Hosmer–Lemeshow test was used to evaluate the goodness of fit of the risk prediction model.

The significance level was set at 0.05. Statistical analyses were performed with the SAS statistical package (version 9.4; SAS Institute, Cary, NC, USA). Given the exploratory observational nature of the study, that was based on data collected during the first COVID-19 wave (February 22 to 10 May 2020; 11 weeks), a sample size calculation was not performed, and all consecutive patients complying with the inclusion criteria were recruited.

## Results

### Study population

In the study period (11 weeks), 137 consecutive patients with acute cerebrovascular diseases were admitted to hospital: 30 (21.9%) patients had COVID-19 infection and represented 2.5% of the 1218 hospitalized COVID-19 patients. Among COVID + patients, 24 (80%) were confirmed COVID cases and 6 (20%) were probable COVID cases. Acute ischemic stroke was the most common stroke type in both COVID + and non-COVID populations (90% versus 83.2%), with no between-group differences in the overall proportion of stroke types (Table 1). Age (median; range min–max: 73; 39–89 vs 79; 36–92), proportion of men (53.3% versus 52.3%), risk factors, comorbidities, and previous antiplatelet/anticoagulant therapy were not different between COVID + and non-COVID stroke populations (Table 1).

**Table 1:** Baseline characteristics of the study population

	Unit	COVID + median (IQR) or *n* (%)	COVID − median (IQR) or *n* (%)	*p*
***Acute cerebrovascular diseases***		(*n* = 30)	(*n* = 107)	
Age	Years	73 (62–80)	78 (66–84)	0.168
Young onset (age < 55 years)	*n* (%)	2 (6.6)	9 (8.4)	0.756
Male/female sex	(%)	53.3	52.3	0.923
**Types of stroke**				0.128
Acute ischemic stroke	*n* (%)	27 (90)	89 (83.18)	
Intracerebral hemorrhage	*n* (%)	1 (3.33)	14 (13.08)	
Cerebral venous thrombosis	*n* (%)	1 (3.33)	0 (0)	
Transient ischemic attack	*n* (%)	1 (3.33)	4 (3.74)	
**Medical history**				
Hypertension	*n* (%)	19 (63.33)	80 (74.77)	0.216
Diabetes	*n* (%)	5 (16.67)	22 (20.56)	0.635
Atrial fibrillation	*n* (%)	6 (20.00)	15 (14.01)	0.403
Smoking	*n* (%)	24 (22.4)	4 (13.33)	0.104
Charlson comorbidity index	*n*	4 (2–5)	4 (3–5)	0.387
Antiplatelet therapy	*n* (%)	7 (23.33)	38 (35.51)	0.209
Anticoagulant therapy	*n* (%)	5 (16.67)	14 (13.08)	0.615
***Acute ischemic stroke***		(*n* = 27)	(*n* = 89)	
**Cerebral vascular territory**				0.0048
Anterior circulation	*n* (%)	16 (59.3)	71 (79.8)	
Posterior circulation	*n* (%)	3 (11.1)	13 (14.6)	
Multiple vascular territories	*n* (%)	7 (25.9)	5 (5.6)	
Unknown	*n* (%)	1 (3.7)	0 (0.00)	
**Large vessel occlusio**n	*n* (%)	33 (37.1)	9 (33.3)	0.773
**NIHSS on admission**	*n*	8 (4–17)	6 (4–11)	0.250
**Recanalization therapies**	*n* (%)	5 (18.5)	34 (38.2)	0.019
**Etiology**				0.323
Large vessel atherosclerosis	*n* (%)	4 (14.8)	16 (18.2)	
Cardioembolic	*n* (%)	12 (48.1)	31 (35.2)	
Small vessel disease	*n* (%)	3 (11.1)	20 (22.7)	
Other definite causes	*n* (%)	1 (3.7)	0 (0.0)	
Cryptogenic	*n* (%)	7 (25.9)	21 (23.9)	

*IQR*, interquartile range; *n*, number; *NIHSS*, National Institute of Health Stroke Scale

### Acute ischemic stroke etiology, severity, and acute treatments

The proportion of acute ischemic lesions in multiple vascular territories (25.9% versus 5.6%; *p* = 0.0048) was higher in COVID + patients than in non-COVID patients, whereas NIHSS on admission, the proportion of large vessel occlusion and etiology did not differ between the two groups (Table 1). COVID + patients with acute ischemic stroke received less commonly acute recanalization treatments (18.5 versus 38.2; *p* = 0.019; Table 1).

### COVID symptoms and timing between COVID symptoms and stroke

**Fig. 1:**
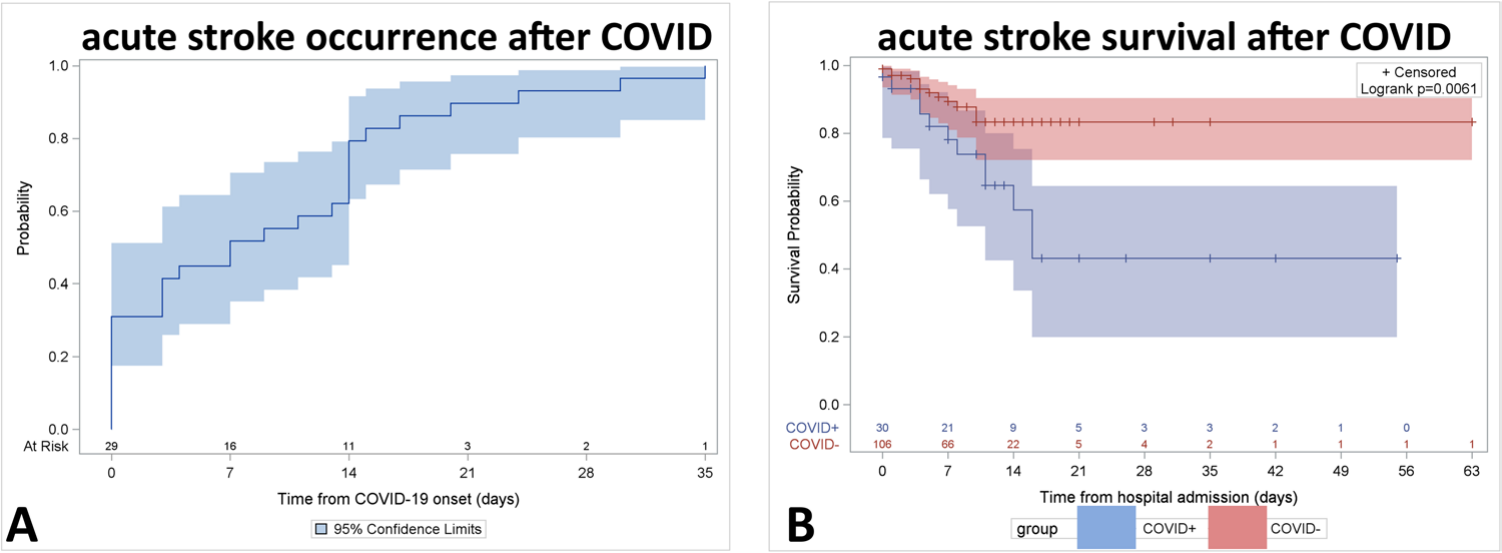
Cumulative incidence (with 95% confidence interval) of stroke occurrence after onset of COVID symptoms (**A**). Survival probability (with 95% confidence intervals) of stroke patients with and without COVID infection after hospital admission (**B**)

The majority of COVID + stroke patients had symptomatic COVID infection (60%) prior to stroke onset, characterized by fever (56.7%) and less commonly by respiratory symptoms (40%), whereas COVID infection was asymptomatic in 40% of stroke patients (Table 2). The cause of hospital admission was acute stroke in 77% of cases and COVID-related symptoms in 23%. The median time between onset of COVID-related symptoms and stroke was 7 days; stroke onset occurred within the first week of COVID symptom onset in approximately 50% of patients, in the second week in 30%, and between the third and fourth week in < 20% (Fig. 1A).

**Table 2:** Pre-stroke clinical presentation of COVID-19

	Unit	COVID + stroke patients (*n* = 30) median (IQR) or *n* (%)
**Symptomatic COVID infection prior to stroke onset**	*n* (%)	18 (60.0)
Fever prior to stroke onset	*n* (%)	17 (56.7)
Respiratory symptoms prior to stroke onset	*n* (%)	12 (40.0)
**Asymptomatic COVID infection prior to stroke onset**	*n* (%)	12 (40.0)
Time between onset of COVID symptoms and stroke	Days	7 (0–14)
**Cause of hospital admission**		
Stroke	*n* (%)	23 (76.7)
COVID-related symptoms	*n* (%)	7 (23.3)

### Infectious disease and respiratory phenotype of stroke patients

On admission, COVID + stroke patients presented the typical COVID-associated alterations of lymphocytes (1210 versus 1700 per mcL; *p* = 0.015), D-dimer (2030 versus 466 ng/mL; *p* = 0.006), fibrinogen (510 versus 312 mg/dL; *p* = 0.003), C-reactive protein (4.13 versus 0.37 mg/dL; *p* < 0.0001), and lactate dehydrogenase (330 versus 263 U/L; *p* = 0.045), compared to non-COVID stroke patients (Table 3). Interstitial pneumonia occurred in the majority of COVID + stroke patients (70%), whereas it was absent in non-COVID stroke patients. Deep venous thrombosis and pulmonary embolism were rare complications (< 10%) in both groups. Non-COVID infections, including bacterial pneumonia and urinary tract infections, were more common in non-COVID stroke patients (57% versus 15%; *p* = 0.0004). Respiratory failure was significantly more common in COVID + stroke patients, compared to non-COVID stroke patients, with need of supplemental oxygen in 77% versus 29% of cases (*p* < 0.0001; Table 3). Nonetheless, both rate of ICU admission and use of mechanical ventilation did not differ between COVID + and non-COVID stroke patients (13.3% versus 10.3%; 6.7% versus 10.3%, respectively).

**Table 3:** Infectious disease characteristics, respiratory support, and in-hospital mortality of stroke patients

	Unit	COVID + (*n* = 30) median (IQR) or *n* (%)	COVID − (*n* = 107) median (IQR) or *n* (%)	*p*
**Blood tests on admission**				
Lymphocytes	per mcL	1021 (770–1730)	1700 (1250–2160)	0.015
D-dimer	ng/mL	2030 (632–5043)	466 (345–822)	0.006
Fibrinogen	mg/dL	513 (331–567)	312 (270–377)	0.003
C-reactive protein	mg/dL	4.13 (0.62–12.6)	0.37 (0.16–0.82)	< 0.0001
LDH	U/L	330 (261–446)	263 (184–306)	0.045
**Interstitial pneumonia**	*n* (%)	21 (70.00)	0 (0.00)	< 0.0001
**Deep venous thrombosis**	*n* (%)	2 (6.67)	2 (1.87)	0.1678
**Pulmonary embolism**	*n* (%)	3 (10.00)	4 (3.73)	0.177
**Non-COVID infections**	*n* (%)	4 (15.38)	31 (57.41)	0.0004
**Respiratory support**				< 0.0001
Breathing room air	*n* (%)	7 (23.33)	76 (71.03)	
Supplemental oxygen mask	*n* (%)	17 (56.67)	16 (14.95)	
CPAP	*n* (%)	4 (13.33)	4 (3.74)	
Mechanical ventilation	*n* (%)	2 (6.67)	11 (10.28)	
**Hospitalization**	Days	11 (5–16)	8 (5–12)	0.266
**ICU admission**	*n* (%)	4 (13.33)	11 (10.28)	0.509
**In-hospital mortality**	*n* (%)	12 (40.00)	13 (12.2)	0.0005
**Cause of death**				0.156
Stroke-related	*n* (%)	4 (33.33)	9 (69.23)	
Respiratory failure	*n* (%)	7 (58.33)	3 (23.08)	
Other causes	*n* (%)	1 (8.33)	1 (7.69)	

*IQR*, interquartile range; *n*, number; *LDH*, lactate dehydrogenase; *CPAP*, continuous positive airway pressure; *ICU*, intensive care unit

### Neurovascular and infectious disease determinants of in-hospital mortality

In-hospital mortality was significantly higher in COVID + stroke patients than in non-COVID stroke patients (40% versus 12.2%; *p* = 0.0005) and in most cases occurred within 7–10 days of admission (Table 3 and Fig. 1B). Multivariable analysis showed that in-hospital mortality was independently associated with symptomatic interstitial pneumonia (adjusted odds ratio: 6.7; 95% CI: 2.0–22.5; *p* = 0.002) and, to a lesser extent, with NIHSS on admission (adjusted odds ratio for each additional point: 1.1; 95% CI: 1.03–1.2; *p* = 0.007) and recanalization therapies (adjusted odds ratio: 0.2; 95% CI: 0.04–0.98; *p* = 0.046). Conversely, age, Charlson comorbidity index, and involvement of multiple vascular territories did not influence in-hospital mortality, after adjustment for confounders (Table 4). The Hosmer–Lemeshow test showed that this risk prediction model was well calibrated, with no evidence of a poor fit (*p* = 0.7161).

**Table 4:** Univariable and multivariable analyses of the effect of selected indicators on survival in the study population

	Survivors (*n* = 112) median (IQR) or *N* (%)	Non-survivors (*n* = 25) median (IQR) or *N* (%)	Crude OR (95% CI)	*p* value	Adjusted OR (95% CI)	Adjusted *p* value
Age (years)	76 (65–83)	81 (72–85)	1.03 (0.99–1.08)	0.075	1.034 (0.97–1.09)	0.281
NIHSS at stroke onset	6 (4–11)	12 (7–19)	1.08 (1.02–1.14)	0.004	1.09 (1.02–1.17)	0.007
Charlson index	4 (3–5)	4 (3–5)	1.12 (0.90–1.38)	0.285	1.00 (0.72–1.37)	0.997
Multiple vascular territories (acute ischemic stroke)	7 (6.3)	6 (24.0)	4.74 (1.43–15.65)	0.011	2.87 (0.71–11.59)	0.137
Recanalization therapies (intravenous rt-PA and/or mechanical thrombectomy)	36 (32.1)	3 (12.0)	0.29 (0.08–1.02)	0.055	0.19 (0.04–0.97)	0.046
Symptomatic COVID-19 pneumonia*	8 (7.1)	10 (40.0)	8.17 (2.99–25.41)	0.0001	6.71 (2.00–22.45)	0.002

*IQR*, interquartile range; *N*, number; *OR*, odds ratio; *CI*, confidence intervals; *NIHSS*, National Institute of Health Stroke Scale.*COVID-associated interstitial pneumonia requiring supplemental oxygen, continuous positive airway pressure (CPAP), or mechanical ventilation

## Discussion

Although the causal relationship between COVID-19 and acute stroke is a highly controversial issue [14, 15], multiple studies agree to indicate an excess mortality of COVID + stroke patients compared to non-COVID stroke patients [16, 17].

COVID-related clinical features and respiratory involvement in patients with concomitant stroke and SARS-CoV-2 infection are poorly characterized, despite their potential relevance on neurological outcome. Moreover, most large studies published on this topic are systematic reviews of pooled case series, with an inherent risk of bias in case ascertainment and data collection [18].

In this study, we investigated the neurovascular and infectious disease characteristics of consecutive patients with acute stroke, with and without COVID-19 infection, admitted to a stroke hub during the first COVID wave in Lombardy, Italy, and their effect on in-hospital mortality and cause of death. The COVID + stroke population represented 2.5% of the total COVID-19 population and 22% of the total stroke population admitted to the San Gerardo Hospital in the study period, in agreement with previous studies conducted in areas hit hardest by the pandemic [19, 20]. Our results showed that COVID + stroke patients did not differ from non-COVID ones in terms of pre-stroke status and neurovascular phenotype, even though an apparent excess of ischemic versus hemorrhagic stroke emerged (which was not statistically significant with our sample size). COVID + patients showed a larger proportion of acute ischemic stroke in multiple vascular districts, compared to non-COVID ones. This finding is in agreement with previous reports, suggesting that embolic etiology is prevalent in COVID + patients [21]. However, our results showed a remarkably similar stroke severity, proportion of large vessel occlusion, and cryptogenic etiology in the two groups, in contrast with previous series [8–10]. Notably, a lower access to recanalization therapies was observed in COVID + patients with acute ischemic stroke than in non-COVID ones, due to either delayed presentation or longer delay in intra-hospital management of acute stroke. This result is in line with other published cohort studies [22].

Our findings indicate that the majority of COVID + stroke patients had symptomatic COVID-19 infection approximately 1 week before stroke onset and developed interstitial pneumonia associated with respiratory failure in more than three-quarters of cases (resulting in a 2.5-fold increased rate of respiratory failure compared to non-COVID patients). Although COVID + stroke patients developed pneumonia and respiratory failure more frequently than non-COVID ones, admission to ICU was similar in the two groups. Moreover, the use of CPAP in COVID + stroke patients was approximately a half of that reported for non-stroke COVID patients [23].

In accordance with previous studies, our results confirmed an exceedingly higher mortality in COVID + stroke patients (more than a threefold increase) than in non-COVID ones. Furthermore, the strongest driver of mortality was symptomatic COVID pneumonia, which predicted in-hospital mortality independently from well-established stroke-related factors associated with survival, such as stroke severity at presentation [24] and recanalization therapies [25, 26].

Two specific factors contributed to poor outcome in COVID + stroke patients during the first COVID-19 wave: (i) the combination of two serious conditions, i.e., acute stroke and COVID-19 pneumonia, that proved to be life-threatening and required multi-disciplinary management; (ii) a limited access to non-invasive and invasive ventilation of COVID + stroke patients in the setting of saturated hospital capacity.

The study has strengths and limitations. The first strength is the consecutive enrollment of concurrent COVID + and non-COVID patients with acute stroke admitted to any hospital department of a large stroke hub, generating a representative cohort and reducing sampling bias. The second strength is the multi-disciplinary nature of this study, with the contribution of both stroke neurologists and infectious disease specialists that allowed systematic collection of infectious disease and respiratory data not reported in previous works on this topic. The major limitation is the small sample size that leads to a lack of power for detecting some potential associations between risk factors and outcomes that were investigated in this study. Post hoc power calculations for selected outcomes are shown in the Online Resource 1. However, our results are supported by the remarkable consistency with other larger cohorts on the baseline characteristics of the study population. Although further confirmatory studies are required, our findings indicate that the infectious disease phenotype, characterized by a high rate of symptomatic interstitial pneumonia, was the major driver of in-hospital mortality of COVID + stroke patients.

## Supplementary Information

Below is the link to the electronic supplementary material.

Supplementary file1 (PDF 82 KB)
