# Supplementary material for: Neurovascular and infectious disease phenotype of acute stroke patients with and without COVID-19
Source: Neurol Sci. 2022 May 23;43(8):4619–25. doi: 10.1007/s10072-022-06133-5 (PMC9125531; doi:10.1007/s10072-022-06133-5)
Supplement: Supplementary file 1 — Supplementary file1 (PDF 82 KB) [file 10072_2022_6133_MOESM1_ESM.pdf]

Neurovascular and infectious disease phenotype of acute stroke patients with and without COVID-19

Simone Beretta[a,b], Francesca Iannuzzi[c], Susanna Diamanti[a], Elisa Bianchi[d], Luca D'Urbano[a], Colella Elisa[c], Alban Rugova[c], Carlo Morotti Colleoni[a], Ettore Beghi[d], Paolo Bonfanti[c] and Carlo Ferrarese[a,b]

[a] Department of Neurology, San Gerardo Hospital ASST Monza, University of Milano Bicocca, Monza, Italy
[b] NeuroMi (Milan Centre for Neuroscience), Milan, Italy
[c] Department of Infectious Diseases, San Gerardo Hospital ASST Monza, University of Milano Bicocca, Monza, Italy
[d] Department of Neuroscience, Istituto di Ricerche Farmacologiche Mario Negri IRCCS, Milan, Italy

Corresponding Author: Simone Beretta, MD, PhD, email: simone.beretta@unimib.it

Journal: **Neurological Sciences**

Online Resource 1

Post-hoc power calculation for in-hospital mortality (primary outcome)
Considering in-hospital mortality in patients with and without symptomatic COVID pneumonia as primary outcome, assuming a type I error of 0.05 and a reference proportion in non-symptomatic COVID pneumonia of 0.071, with a sample size of 137 patients (18 with symptomatic COVID pneumonia and 119 without symptomatic COVID pneumonia), the power to detect an OR of 8.17 is 0.91.

Post-hoc power calculation for ischemic stroke
With a proportion of 90% ischemic strokes in COVID+ patients and 83.2% in COVID-, with a sample size of 30 COVID+ and 107 COVID-, the power to detect this difference as statistically significant with 5% level of significance is 12%.

Post-hoc power calculation for cerebrovascular territories
With a proportion of 25.9% multiple vascular territories in COVID+ patients and 5.6% in COVID-, with a sample size of 30 COVID+ and 107 COVID-, the power to detect this difference as statistically significant with 5% level of significance is 84%.
